# Cognitive impairment and health outcomes in non-dialysis chronic kidney disease: a systematic review and meta-analysis

**DOI:** 10.1093/ckj/sfaf150

**Published:** 2025-05-19

**Authors:** Keegan Guan Ru Lee, Anooj Ghadge, Bhargav Raut, Kristin Veighey, Jay Amin, Giovambattista Capasso, Paul Cockwell, Philip A Kalra, Marion Pepin, Tom Phillips, Maarten Taal, Robert Unwin, Simon D S Fraser

**Affiliations:** School of Primary Care, Population Sciences and Medical Education, University of Southampton, Southampton, UK; Department of Medicine, University Hospital Southampton NHS Foundation Trust, Southampton, UK; School of Primary Care, Population Sciences and Medical Education, University of Southampton, Southampton, UK; Primary Care Research Centre, University of Southampton; School of Clinical and Experimental Sciences, Faculty of Medicine, University of Southampton, Southampton, UK; Biogem Institute for Molecular Biology and Genetics, Airano Irpino, Italy; Department of Renal Medicine, Queen Elizabeth Hospital, Birmingham, UK; Institute of Inflammation and Ageing, University of Birmingham, UK; Donal O'Donoghue Renal Research Centre, Salford Royal Hospital, Northern Care Alliance NHS Foundation Trust, Salford, UK; CESP (INSERM U-1018), Clinical Epidemiology, Paris Saclay University, Villejuif, France; Geriatric department, Ambroise Paré Hospital, Versailles St Quentin University, Boulogne-Billancourt, France; School of Primary Care, Population Sciences and Medical Education, University of Southampton, Southampton, UK; Wessex Kidney Centre, Portsmouth Hospitals University NHS Trust, Portsmouth, UK; Department of Renal Medicine, University Hospitals of Derby and Burton NHS Foundation Trust, Derby, UK; Centre for Kidney Research and Innovation, Academic Unit for Translational Medical Sciences, School of Medicine, University of Nottingham, Nottingham, UK; Department of Renal Medicine, UCL Medical School, London, UK; School of Primary Care, Population Sciences and Medical Education, University of Southampton, Southampton, UK

**Keywords:** chronic kidney disease, cognitive impairment, health outcomes, mortality, self-management

## Abstract

**Background:**

Cognitive impairment is prevalent in individuals with chronic kidney disease (CKD), but its effects on health outcomes remain unclear. While cognitive impairment can affect self-management, its role in CKD has been insufficiently explored. This systematic review aimed to examine the association between cognitive impairment and health outcomes or self-management ability among persons with CKD.

**Methods:**

Searches were performed in June 2024 on Embase, MEDLINE, CINAHL, PsycINFO, Web of Science, PubMed and grey literature databases for longitudinal or cross-sectional studies examining associations between cognitive impairment (using any validated measure) and health outcomes or ability to self-manage in adults with CKD not on kidney replacement therapy. Health outcomes included mortality, kidney disease progression, hospitalization and healthcare utilization, cardiovascular and cerebrovascular events, and health-related quality of life (HRQoL). Risk of bias was assessed using the ROBINS-E (‘Risk of bias in non-randomized studies of exposure’) tool.

**Results:**

Fourteen studies were included. Cognitive impairment was associated with increased all-cause and cardiovascular mortality, higher risk of cardiac arrhythmia, stroke and transient ischaemic attack, lower HRQoL, and higher healthcare utilization. Mixed results were seen in studies examining the association between cognitive impairment and kidney disease progression. No studies with self-management measures as an outcome were identified.

**Conclusions:**

Cognitive impairment is associated with poor health outcomes in persons with CKD, although evidence was limited for some outcomes. No causal link could be established due to potential residual confounding by frailty or shared comorbidities. Further research is required to explore potential causal pathways and the role of cognitive impairment in CKD self-management.

KEY LEARNING POINTS **What was known:**

- Chronic kidney disease (CKD) is associated with a higher risk of cognitive impairment.
- Cognitive impairment affects ability to self-manage in other chronic conditions such as heart failure and chronic obstructive pulmonary disease.
- It is not known how cognitive impairment affects key health outcomes or self-management ability in persons with CKD.

**This study adds:**

- Cognitive impairment is associated with increased mortality, higher rates of arrhythmia and cerebrovascular events, lower health-related quality of life and higher rates of healthcare utilization in persons with CKD.
- However, a causal relationship could not be established due to heterogeneity and lack of sufficient confounder adjustment in some studies.
- No studies assessing the association between cognitive impairment and self-management measures in CKD were identified.

**Potential impact:**

- Screening for cognitive impairment should be considered among persons with CKD to allow earlier management and support for those with cognitive impairment.
- Cognitive impairment should be included to a greater extent in prognostication and planning of current and future management in persons with CKD.
- Further research is required to explore causal pathways and the role of cognitive impairment on self-management among persons with CKD.

## INTRODUCTION

Cognitive impairment is a deficiency in one or more cognitive domains on a standardized neuropsychological assessment [1]. It ranges from mild cognitive impairment (MCI), whereby there is greater than expected cognitive impairment for a person's age and education level but functional ability is retained, to dementia, where there is more significant impairment across multiple cognitive domains resulting in impairment in a person's ability to conduct activities of daily living [1–3]. A meta-analysis in 2024 reported that 32% (95% confidence interval 25%–38%) of persons with CKD not on kidney replacement therapy (KRT) have cognitive impairment [4]. Impaired kidney function, measured by estimated glomerular filtration rate (eGFR) or proteinuria, has been associated with higher risk of both MCI and all-cause dementia, albeit with a low level of certainty [5, 6].

The mechanisms linking CKD and cognitive impairment are multifactorial and may include vascular injury, uraemic toxins, inflammatory mediators, gut dysbiosis, accelerated aging and impairment of the glymphatic system, which is a waste clearance system in the brain that removes metabolites such as tau and β-amyloid—with complex interplay between these processes [1, 7–10]. Several complications of CKD, including metabolic acidosis, renal anaemia, and CKD mineral and bone disorder, may also contribute to the development of cognitive impairment [11–14]. Persons with CKD also commonly have comorbidities that are independent risk factors for cognitive impairment such as hypertension, diabetes, hyperlipidaemia and obesity [12, 15].

Self-management comprises a person's ability to manage symptoms, treatment and lifestyle changes associated with a medical condition. Interventions to improve self-management in CKD can increase patient knowledge, motivation and perceived self-management ability [16, 17]. They may also improve health-related quality of life (HRQoL), reduce CKD-related symptoms and reduce healthcare utilization, although evidence for this is weaker due to variability in interventions and outcomes across studies [16, 17].

Cognitive impairment has been demonstrated to negatively impact self-management in chronic conditions such as heart failure, chronic obstructive pulmonary disease (COPD) and diabetes [18–20]. Effects include impaired decision-making, reduced self-monitoring, and reduced early interaction with healthcare professionals leading to worse complications, increased disability and impaired medication use [18–20]. However, the effect of cognitive impairment on the self-management of CKD remains understudied.

While most research has focused on CKD as a risk factor for developing cognitive impairment, less is known about whether cognitive impairment is an independent risk factor for poorer health outcomes in persons with CKD. This systematic review examines the association between cognitive impairment and health outcomes or self-management ability in individuals with CKD.

## MATERIALS AND METHODS

This study followed the Preferred Reporting Items for Systematic Reviews and Meta-Analyses (PRISMA) guidance [21].

We aimed to identify cross-sectional or cohort studies involving persons with CKD that investigated cognitive impairment as an exposure and health outcomes or self-management as study outcomes. The inclusion criteria for the population were adults with CKD of any stage but excluding those on KRT. Cognitive impairment was defined as a diagnosis of dementia or MCI on health records, or evidence of cognitive impairment using a validated cognitive assessment tool. Health outcomes included all-cause and cardiovascular mortality, progression of kidney disease (as defined by individual studies, including need for KRT), hospitalization and healthcare utilization, cardiovascular and cerebrovascular events, or HRQoL measures. Self-management outcomes included treatment or medical adherence or any patient-reported measures of self-management ability such as the self-care inventory, self-management ability scale or patient activation measure. The PECO-D framework, full inclusion and exclusion criteria, and search strategy are provided in the Supplementary Material section.

The search protocol was registered on PROSPERO (Registration ID: CRD42024547379). A comprehensive search was conducted in June 2024 across multiple databases, including Embase, MEDLINE, CINAHL, PsycINFO, Web of Science and PubMed. Grey literature sources such as Google Scholar, Open Grey, Proquest, TRIP, Bielefeld Academy Search Engine and JISC Library Hub were also reviewed. Additional studies were identified through citation searching.

We de-duplicated records manually and using automation tools (Rayyan and EndNote). Two independent reviewers (K.G.R.L. and A.G.) screened records with disagreements resolved by a third reviewer (S.D.S.F.).

From each study we extracted publication information, study design, participant data, exposure details and outcomes measured with summary statistics. Quality assessment of the included studies was performed at study level using the ‘Risk of bias in non-randomized studies of exposure’ (ROBINS-E) tool [22]. The risk of bias assessment was performed by two independent reviewers (K.G.R.L. and B.R.) with disagreements resolved by a third reviewer (S.D.S.F.).

Study conclusions with summary statistics were grouped by reported study outcomes. For studies with multiple hazard ratios (HRs) for different subgroups, pooled hazard ratios and confidence intervals were calculated [23, 24].

Only all-cause mortality had sufficient studies for meta-analysis. A meta-analysis of aggregate data of summary statistics was performed in R using the meta package with both the fixed and Hartaung–Knapp random effects model. The effect measure was HRs with 95% confidence intervals. Results are presented using a Forest plot. Weighting using both models are displayed. Two measures of heterogeneity, *I*^2^and τ^2^, were calculated to quantify between-study variability. Sensitivity analysis was conducted by performing the meta-analysis excluding studies rated as having a high risk of bias. All other outcomes with reported hazard, risk or odds ratios are presented in a Forest plot without meta-analysis.

The formulas for calculating pooled HRs and the meta-analysis code are provided in the Supplementary Materials section.

## RESULTS

**Figure 1:**
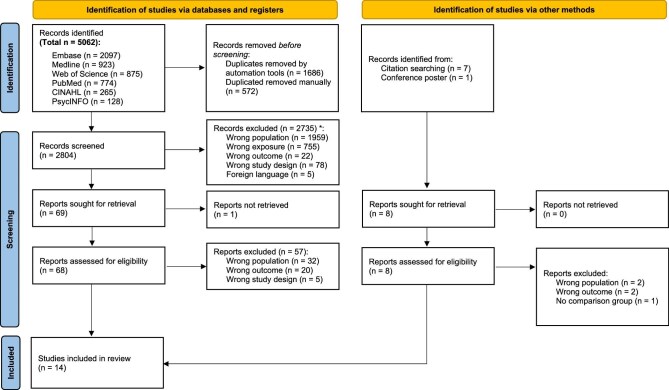
Flow chart of study identification process. Figure adapted from PRISMA 2020 Guidelines (Page MJ 2021). *Some studies had more than one reason for exclusion.

A total of 5062 records were identified from database searching and 2258 were removed via de-duplication. Of 2804 unique records, 2735 failed to meet eligibility criteria on abstract screening alone (Fig. 1). The full text for the remaining 69 records, in addition to 8 identified via other methods, were sought for retrieval. After assessing using the inclusion and exclusion criteria, 14 papers were included in this review [25–38].

### Study characteristics

Table 1 summarizes the characteristics of included studies. Nine (64%) of the included studies were published within the last 5 years, and 10 (71%) were conducted exclusively in North America or Europe. The majority (71%) were prospective cohort studies, with the remainder consisting of three cross-sectional (29%) and one retrospective cohort study (7%). The median follow-up time for cohort studies was 25.6 months [interquartile range (IQR) 38.8 months].

**Table 1:** Summary of study characteristics of included studies in this review.

Authors	Year of publication	Country	Design	Duration (months)^a^	Participant population^c^	Exposure and measure	Number (%) in exposure group^g^	Relevant outcomes
Bai	2018	China	Prospective cohort	28 (19–40)	CKD G3–5, age >80	CI: MMSE	109 (66.9%)	AC and CV mortality
Burrows	2022	USA	Prospective cohort (database)	54 (27–96)	CKD G3	Dementia (ICD code)	49 837 (5.6%)	AC mortality
Chiu	2022	USA	Prospective cohort	116	eGFR 20–70, age 21–75	CI: 3MS	12 (1.4%)	AC mortality, dialysis initiation, hospitalization
Corsonello	2022	Europe	Prospective cohort	22.3 ± 4.9	Age >75 (66.4%)^d^	CI: MMSE	183 (7.7%)	AC mortality
Di Rosa	2020	Italy	Prospective cohort	12	Age >65 (57.3%)^d^	CI: MMSE	318 (49.2%)	1-year AC mortality
Faruque	2013	Canada	Prospective cohort (database)	11 (3–27)	CKD G5	Dementia (ICD code)	1297 (16.4%)	AC mortality, dialysis initiation
Greinert	2023	Germany	Cross-section	n/a	CKD G3–5	CI: 3MS, TMT (A&B) and domain tests^e^	37 (59.7%)	HRQoL
Jayanti	2016	UK	Prospective cohort	12^b^	CKD G5	CI: 3MS, TMT (A&B), meta-cognition	15 (7.2%)	Dialysis modality choice
Kurella	2016	USA	Prospective cohort	73 (49–89)	eGFR 20–70, age 21–75	CI: 3MS	524 (13.5%)	Kidney disease progression/dialysis initiation
Lu	2022	Taiwan	Prospective cohort	24^b^	T2DM (77.9 ± 21.9)^d^	CI: MMSE	486 (14.3%)	Kidney disease progression
Merlino	2024	Global	Retrospective cohort	23.2	CKD G3–5, age 18–70	Dementia or MCI (ICD code)	8170 (50%)	AC mortality, cardiovascular events, cerebrovascular events
Raphael	2012	USA	Prospective cohort	72–100^b^	Age >60 (21.5%)^d^	CI: abbreviated MMSE	992 (19.5%)	AC mortality
Sheets	2022	USA	Cross-section	n/a	CKD G3–5, age ≥45	CI: domain tests^f^	202 (48.1%)	Medication adherence
Thancharoen	2020	Thailand	Cross-section	n/a	CKD G3–5	CI: MMSE	60 (15.8%)	HRQoL, healthcare utilization

a Duration is the actual follow-up duration (in months), unless otherwise stated, and where available expressed as median (IQR) or mean ± standard deviation.

b Duration is the planned follow-up duration (in months) as stated in the study protocol, listed when actual follow-up times were not reported.

c Unless otherwise state, inclusion criteria for age was adults ≥18 years.

d For studies which did not recruit CKD patients exclusively, a broad description of the participant population was stated. The proportion of patients with CKD G3–5 or the mean eGFR is given in parentheses.

e Domain tests included the Digital Symbol Test (DST), Line Tracing Test (LTT) and Serial Dotting Test (SDT).

f Domain tests included Hopkins Verbal Learning Test-Revised (HVLTR), Brief Visuospatial Memory Test-Revised (BVMTR), Symbol Digit Modalities Test (SDMT), Color Trail Tests (CTT1&2), Controlled Oral Word Association Test (COWAT) and Animal naming section of 3MS.

g Number of participants in exposure group expressed as percentage of total study population in parenthesis.

AC, all-cause (mortality); CI: cognitive impairment; CV, cardiovascular (mortality); ICD, International Classification of Diseases; T2DM, type 2 Diabetes Mellitus.

The total number of participants across all included studies was 934 221 with a median sample size of 1747.5 (IQR 4396). The median number of participants with cognitive impairment was 260 (IQR 802.75) Most studies (86%) had a mean or median participant age of >60 years old. In eight studies (57%), all participants had CKD Stage G3A to G5 (eGFR <60 mL/min/1.73 m^2^), of which two studies (14%) only had participants with CKD Stage G5 (eGFR <15 mL/min/1.73 m^2^). Out of the four studies including non-CKD participants, two had at least 50% of participants with CKD Stage G3A to G5 (eGFR <60 mL/min/1.73 m^2^).

In 11 studies (79%), the exposure was cognitive impairment identified using cognitive assessment tools rather than a clinical diagnosis of dementia or cognitive impairment from medical records. The most used cognitive tests in these studies were the Mini-Mental State Examination (MMSE) (45%), Modified Mini-Mental State Examination (3MS) (36%) and Trail Making Tests (TMT) (27%). Prevalence of cognitive impairment in participants varied: in nine studies (64%), ≤20% of total participants had cognitive impairment; in two studies (14%), 21%–49% of total participants had cognitive impairment; and in three studies (21%), ≥50% of total participants had cognitive impairment.

### Study outcomes

A summary of study conclusions and summary statistics is provided in Table 2.

**Table 2:** Study conclusions and summary statistics.

Study	Participant population	Number in exposure group	Summary statistic	Subgroup statistics	Conclusion
All-cause mortality					
Bai	CKD G3–5, age >80	109 (66.9%)	Categorical:	MMSE 19–23: HR 3.30 (0.77–14.16)	CI (MMSE) associated with increased all-cause mortality, with higher risk with increasing CI severity
			HR 5.32 (1.95–14.49)^c^	MMSE ≤19: HR 8.18 (2.05–32.54)	
			Continuous:^d^		
			1-point MMSE decrease: HR 1.41 (1.15–1.72)		
Burrows	CKD G3	49 837 (5.6%)	HR 1.70 (1.68–1.72)	Age 18–44: HR 3.09 (1.57–6.08)	Dementia diagnosis associated with increased all-cause mortality; association most pronounced in the youngest subgroup (age 18–44)
				Age 45–64: HR 1.39 (1.31–1.47)	
				Age 65–84: HR 1.71 (1.68–1.74)	
				Age 85–100: HR 1.69 (1.65–1.73)	
Chiu	eGFR 20–70, Age 21–75	12 (1.4%)	HR 0.72 (0.32–1.64)		No association between CI (3MS) and all-cause mortality
Corsonello	Age >75 (66.4%)^a^	183 (7.7%)	HR 1.21 (0.65–2.26)		No association between CI (MMSE) and all-cause mortality
Di Rosa	Age >65 (57.3%)^a^	318 (49.2%)	HR 1.64 (0.97–2.78)^c^	eGFR <30: HR 3.12 (1.26–7.77)	CI (MMSE) associated with increased all-cause mortality in CKD G4–5 (eGFR <30) only
				eGFR 30–44.9: HR 1.08 (0.42–2.77)	
				eGFR 45–59.9: HR 1.29 (0.53–3.13)	
				eGFR >60: HR 0.56 (0.10–2.95)	
Faruque	CKD G5	1297 (16.4%)	HR 2.18 (2.01–2.36, *P* < .05)		Dementia diagnosis associated with increased all-cause mortality
Merlino	CKD G3–5, age 18–70	8170 (50%)	HR 1.3 (1.50–1.76, *P* < .001)		Dementia/MCI diagnosis associated with increased all-cause mortality
Raphael	Age >60 (21.5%)^a^	992 (19.5%)	HR 1.42 (1.03–1.96)^c^	Cognitive score 0–5: HR 2.02 (1.11–3.67)	CI (abbreviated MMSE) associated with increased all-cause mortality in group with lowest cognitive score. Trend suggests increasing mortality with decreasing score, but not statistically significant
				Cognitive score 6–8: HR 1.24 (0.85–1.80)	
				Cognitive score 9–10: HR 1.14 (0.80–1.62)	
Cardiovascular mortality					
Bai	CKD G3–5, age >80	109 (66.9%)	Categorical:	MMSE 19–23: HR 6.55 (0.69–62.27)	CI (MMSE) associated with increased cardiovascular mortality, with higher risk with increasing CI severity
			HR 9.93 (2.07–47.70)^c^	MMSE ≤19: HR 14.72 (1.65–131.16)	
			Continuous:^d^		
			1-point MMSE decrease: HR 1.64 (1.20–2.27)		
Kidney disease progression and KRT initiation					
Chiu	eGFR 20–70, age 21–75	12 (1.4%)	HR 2.17 (0.98–4.8)		No association between CI (3MS) and dialysis initiation
Lu	T2DM (77.9 ± 21.9)^a^	486 (14.3%)	HR 1.25 (1.07–1.44)^c^	MMSE 19–23: HR 1.17 (0.97–1.42)	Severe CI (MMSE ≤19) associated with increased risk of CKD progression, but not moderate CI (MMSE 19–23)
				MMSE ≤19: HR 1.34 (1.06–1.67)	
Kurella	eGFR 20–70, age 21–75	524 (13.5%)	KRT: HR 1.07 (0.87–1.30)		No association between CI (3MS) and KRT or composite outcome (KRT or 50% eGFR reduction) in adjusted models
			KRT or eGFR reduction: HR 1.06 (0.89–1.27)		
Faruque	CKD G5	1297 (16.4%)	HR 0.36 (0.27–0.47, *P* < .05)		Dementia diagnosis associated with lower likelihood of initiating KRT
Dialysis modality choice					
Jayanti	CKD G5	15 (7.2%)	Objective measures:		Objective measures of CI (3MS, TMT) had no statistically significant association with choice of modalityLower meta-concentration score associated with lower likelihood of choosing self-care modality
			3MS (per 10 score decrease): OR 1.59 (0.86–2.96, *P* .14)^e^		
			TMT A (per 10 s decrease): OR 0.91 (0.79–1.05, *P* .21)^e^		
			TMT B (per 10 s decrease): OR 0.96 (0.90–1.02, *P* .16)^e^		
			Subjective measures (metacognition):		
			Meta-concentration (per unit decrease): OR 1.20 (1.05–1.37, *P* .08)^e^		
			Metamemory (per unit decrease): OR 1.01 (0.93–1.10, *P* .80)^e^		
Hospitalization and healthcare utilization					
Chiu	eGFR 20–70, age 21–75	12 (1.4%)	HR 1.35 (0.56–3.26), C-statistic 0.64		No association between CI (3MS) and all-cause hospitalisation
Thancharoen	CKD G3–5	60 (15.8%)	ED visits: RR 3.47 (1.45–8.29)	OP visits:	CI (MMSE) increased the number of ED visits for CKD 3–5, OP visits for CKD 4–5 and hospitalization for CKD 5^f^
			OP visits: RR 1.08 (0.96–1.21)	CKD 4: RR 1.1 (1.0–1.2)	
			Hospitalization: RR 1.43 (0.72–2.83)	CKD 5: RR 1.3 (1.1–1.4)	
				Hospitalization:	
				CKD 5: RR 2.3 (1.3–2.41)	
Cardiovascular events					
Merlino	CKD G3–5, age 18–70	8170 (50%)	Ischaemic heart disease: HR 1.10 (0.98–1.23)		Dementia/MCI diagnosis associated with increased risk of arrhythmia, but not ischaemic heart disease
			Arrhythmia: HR 1.26 (1.14–1.40, *P* < .05)		
Cerebrovascular events					
Merlino	CKD G3–5, Age 18–70	8170 (50%)	Stroke: HR 1.89 (1.67–2.14, *P* < .001)		Dementia/MCI diagnosis associated with increased risk of both stroke and TIAs
			TIA: HR 1.86 (1.49–2.32, *P* < .001)		
Quality of life					
Greinert	CKD G3–5	37 (59.7%)	Median for PCS: 29.9 (20.8–35.4) in CI group vs 41.2 (34.9–48.6) in non-CI group, *P* .001		CI (Composite score) associated with poorer PCS of QoL (SF-36) but no significant difference in MCS
			Median for MCS: 48.3 (38.4–56.4) in CI group vs 52.4 (42.4–57.6) in non-CI group, *P* .545		
Thancharoen	CKD G3–5	60 (15.8%)	Overall QoL score:		CI (MMSE) associated with lower overall QoL (EQ-5D-5L). Participants with CI are more likely to report issues with all EQ-5D-5L domains except for pain
			Mean score in CI 0.78 (SD = 0.16) vs non-CI 0.85 (SD = 0.18), *P* < .003		
			QoL domains:		
			Mobility: OR 2.00 (1.05–3.85, p0.35)^g^		
			Self-care: OR 3.45 (1.79–6.67, *P* < .001)^g^		
			Usual activities: OR 2.27 (1.22–4.17, *P* .009)^g^		
			Pain: OR 1.18 (0.54–2.17, *P* .61)^g^		
			Anxiety/depression: OR 2.56 (1.39–4.55, *P* .002)^g^		
Adherence					
Sheets	CKD G3–5, age ≥45	202 (48.1%)	OR: 0.18 (0.061–0.54, *P* .002)^h^		Participants with CI (multiple tests) had better self-reported medication adherence

a For studies which did not recruit CKD patients exclusively, a broad description of the participant population was stated. The proportion of patients with CKD G3–5 or the mean eGFR is given in parentheses.

b Number of participants in exposure group expressed as percentage of total study population in parenthesis.

c These statistics were not reported in the original paper and were calculated by combining reported HRs in order to create a summary statistics for meta-analysis.

d Study reported this as HR for every 1-point increase in MMSE.

e Odds ratio >1 means a lower chance of selecting a self-care modality choice (home haemodialysis or peritoneal dialysis).

f No statistically significant association between both OP visits and hospitalization with CKD 3–5.

g Odds ratio >1 means a higher likelihood of reporting poor outcome (i.e. issues with domain) in comparison with participants with normal cognition. Odds ratio was inverted from study-reported odds ratio for ease of comparison with other outcomes.

h Odds ratio >1 means a higher likelihood of poor medication adherence in participants with cognitive impairment. Odds ratio was inverted from study-reported odds ratio for ease of comparison with other outcomes.

CI, cognitive impairment; ED, emergency department; MCS, mental component score; OR, odds ratio; PCS, physical component score; RR, relative risk.

#### Meta-analysis of all-cause mortality

**Figure 2:**
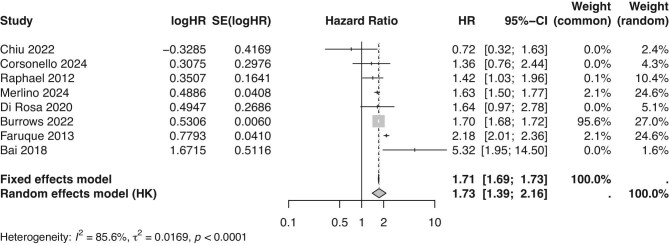
Forest plot showing the results of fixed and random effects of meta-analysis of all-cause mortality.

Meta-analysis of eight studies showed that cognitive impairment was associated with a higher risk of all-cause mortality (random effects model: HR 1.73, 95% confidence interval 1.39–2.16) (Fig. 2). However, there was significant heterogeneity between studies (*I*^2 ^= 85.6%). A sensitivity analysis excluding two studies with high risk of bias showed no substantial change (HR 1.78, 95% confidence interval 1.40–2.26) (Supplementary data, Fig. S6). Two studies found that increased severity of cognitive impairment was associated with increased mortality risk [25, 36]. One study showed that the effect of cognitive impairment was strongest in participants aged 18–44 years compared with those over 45 years, however this conclusion could not be compared with other studies as no other study performed subgroup analysis by age [26]. In one study that analysed patients by eGFR group, the effect of cognitive impairment on increased mortality risk was only seen in those with eGFR <30 mL/min/1.73 m^2^ [29]. However, although no other study performed a formal subgroup analysis for eGFR, three other studies with mean participant mean eGFR ≥45 mL/min/1.73 m^2^ still showed an association between cognitive impairment and increased mortality risk [25, 26, 35].

#### Narrative synthesis of other outcomes

**Figure 3:**
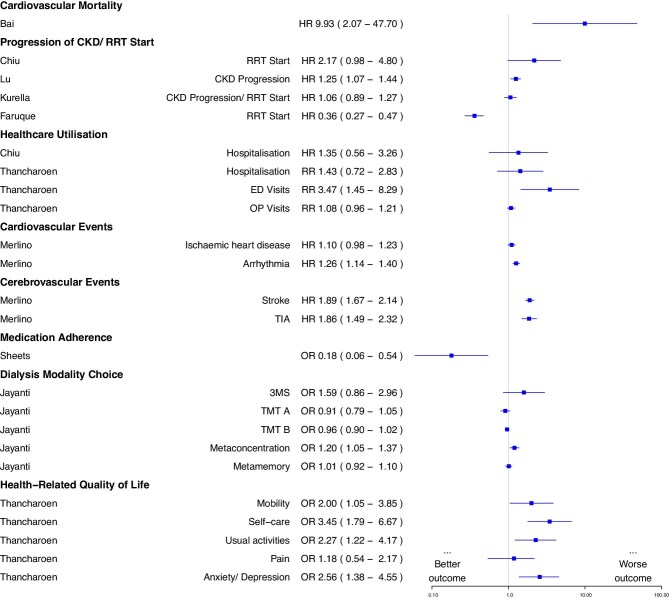
Forest plot showing summary statistics for all other outcomes with reported hazard, risk and odds ratios. Hazard ratio >1 and relative risk >1 reflects higher likelihoods in the group with cognitive impairment. Odds ratio >1 for Medication Adherence outcome reflects poorer medication adherence. Odds ratio >1 for Dialysis Modality Choice reflects higher likelihood of choosing a non-self-care dialysis modality if there was a lower (worse) cognitive score. Odds ratio >1 for HRQoL reflects higher likelihood of reporting a poor outcome (i.e. issues with domain) in comparison with participants with normal cognition.

Figure 3 is a visual summary of all other outcomes with reported hazard, risk or odds ratios. Cognitive impairment was associated with higher cardiovascular mortality risk (*n* = 1), and higher risk of developing cardiac arrhythmias (*n* = 1), strokes and transient ischaemic attack (TIA) (*n* = 1).

**Figure 4:**
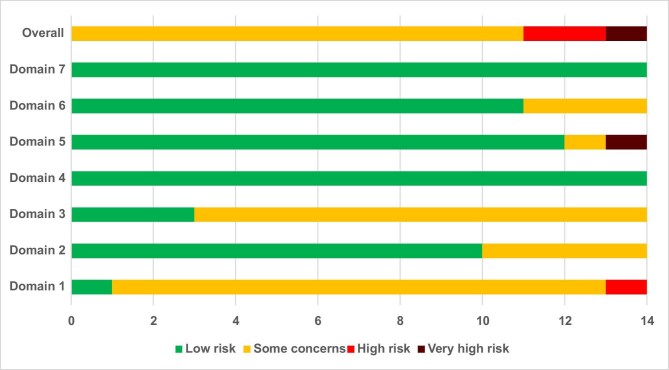
Summary of risk of bias assessment. Domain 1: risk of bias due to confounding. Domain 2: risk of bias arising from measurement of the exposure. Domain 3: risk of bias in selection of participants into the study (or into the analysis). Domain 4: risk of bias due to post-exposure interventions. Domain 5: risk of bias due to missing data. Domain 6: risk of bias arising from measurement of the outcome. Domain 7: risk of bias in selection of the reported result.

There were mixed results in four studies examining the association between cognitive impairment and kidney disease progression including KRT initiation. Cognitive impairment was associated with increased risk of kidney disease progression alone in one study, but in the remaining studies that had either KRT or a composite KRT/kidney disease progression outcome, there was either no association (*n* = 2) or cognitive impairment was associated with lower rates of KRT initiation (*n* = 1).

Cognitive impairment was not associated with an overall increased risk of hospitalization (*n* = 2), except in the CKD G5 cohort of one study [38]. However, cognitive impairment was associated with increased healthcare utilization, showing higher emergency department and outpatient visits at different levels of CKD severity.

In two cross-sectional studies, cognitive impairment was associated with lower HRQoL. In one study that used the EuroQoL-5 Dimension-5 Level (EQ-5D-5L) measure, overall HRQoL was reduced [38]. In the other study, the 36-item short form health survey (SF-36) was used and found a statistically significant association for the physical but not the mental component score [31].

A small study examined the effect of cognition on dialysis modality choice and showed that participants who rated their concentration abilities as poor were more likely to choose a non-self-care modality [32]. However, there was no association found between objective measures (3MS, TMT) and dialysis modality choice.

One cross-sectional study found cognitive impairment was associated with improved self-reported medication adherence, even after sensitivity analysis to limit the sample to participants who self-managed their medications [37].

### Quality assessment

Eleven studies (79%) were rated as having ‘some concerns’ of bias, two (14%) had ‘high risk of bias’ and one (7%) had ‘very high risk of bias’. Most concerns arose in domains measuring risks of bias due to confounding variables, measurement of the exposure and selection of participants (Fig. 4).

Thirteen studies (93%) adjusted for age and sex, and 10 studies (71%) adjusted for at least one comorbidity. However, only six studies (43%) adjusted for a measure of renal function (either eGFR or proteinuria). Several cardiovascular risk factors were also adjusted for in some studies: blood pressure or hypertension diagnosis (57%), body mass index (50%), diabetes (50%), smoking (43%), alcohol use (29%) and serum cholesterol levels (21%). The median number of cardiovascular risk factors adjusted per study was 3 (IQR 1.25–5).

Risk of bias assessments for individual studies and list of adjusted confounders can be found in the Supplementary  Materials section.

## DISCUSSION

### Summary of main findings

This systematic review found that cognitive impairment in individuals with non-dialysis CKD is linked to higher mortality, increased risk of arrhythmia and cerebrovascular events, reduced HRQoL, and greater healthcare utilization. There were conflicting results from studies examining the association between cognitive impairment and kidney disease progression or rates of KRT initiation. There were no studies examining the effect of cognitive impairment on self-management measures.

The meta-analysis showing that cognitive impairment was associated with increased all-cause mortality came from a heterogenous group of studies, with the strength of the association primarily driven by three studies with relatively large sample sizes that identified patients with cognitive impairment through clinical coding in health databases. While this may introduce potential bias as the presence of cognitive impairment depended on the accuracy of coding patients, it likely underestimated the effect of cognitive impairment if some patients remain undiagnosed or uncoded.

It was difficult to compare the four studies looking at cognitive impairment and kidney disease progression due to variation in their patient populations, measurement of cognitive impairment and definition of kidney disease progression. One study showed that patients with a dementia diagnosis were less likely to initiate KRT. However, this study only included patients with CKD G5 and likely reflects that patients with dementia were poorer candidates for initiating KRT, rather than the fact they did not clinically require KRT.

Unexpectedly, one study found that participants with cognitive impairment had better medication adherence. However, this finding should be interpreted cautiously as it relied on a self-reported medication adherence questionnaire.

No studies were found investigating the association between cognitive impairment and self-management measures in CKD. However, dialysis modality choice may serve as a proxy indicator for self-management, given that self-care modalities such as home haemodialysis or peritoneal dialysis involve a high degree of patient or carer involvement. In one included study, participants who self-rated their ability to concentrate as poor were less likely to choose a self-care modality [32].

### Mechanisms linking cognitive impairment and health outcomes

Executive function, attention and memory domains may be affected earlier in cognitive impairment associated with CKD [39, 40]. This could negatively impact self-management thus leading to poorer health outcomes. However, no studies examined this relationship. Systematic reviews in comparable chronic conditions such as COPD, heart failure and diabetes suggest that the presence of cognitive impairment impairs self-management ability [18–20]. Furthermore, systematic reviews on chronic conditions including COPD, asthma, bronchiectasis, heart failure, stroke and osteoarthritis also suggest that improved self-management may improve HRQoL and reduce mortality rates, although evidence is weak due to variation in cognitive measures, interventions and outcomes assessed [41–43]. However, to our knowledge there was no study that examined a link between cognitive impairment, self-management and health outcomes in a single cohort of participants.

CKD and cognitive impairment share common risk factors including hypertension, diabetes, hyperlipidaemia and obesity, all of which are also related to poorer health outcomes [12, 15]. As most included studies did not adjust for all these factors, it is possible that cognitive impairment is not an independent risk factor for health outcomes, but rather poorer outcomes are due to these underlying cardiovascular risk factors.

Alternatively, cognitive impairment may just be an indicator of overall frailty and multi-morbidity. Corsonello *et al*. included measurements of handgrip strength, physical performance and dependency in basic activities of daily living. This study provided the most thorough adjustment for frailty markers and did not find that cognitive impairment was associated with increased mortality. However, it only included a population older than 75 years old and given that no other studies adjusted for frailty in this way, drawing definitive conclusions is challenging. Other potential mediators for the association between cognitive impairment and health outcomes may include psychosocial factors, increased hospitalization and other comorbidities.

### Strengths and limitations

This is the first systematic review that examined the association between cognitive impairment and health outcomes in CKD. We utilized a comprehensive search strategy and adhered to PRISMA guidelines throughout.

Our analysis was limited by a paucity of relevant papers, highlighting a need for further studies that encompass the full spectrum of CKD. Another limitation is the heterogeneity of studies included in this review. There was significant variability in study populations in terms of size, age and CKD stage, measurement and degree of cognitive impairment, and in health outcomes assessed. Except for all-cause mortality, most other outcomes were only examined in two studies or fewer. Additionally, the meta-analysis performed on all-cause mortality was heavily weighted by the studies utilizing healthcare databases, as these studies included large numbers of participants.

Three studies were rated as having ‘high’ or ‘very high’ risk of bias [27, 29, 31]. These were retained due to the limited number of studies, and sensitivity analysis did not substantially alter results. Nonetheless, their inclusion may reduce the certainty of the findings.

### Implications for clinical practice

Individuals with both CKD and cognitive impairment generally experience worse health outcomes than those with CKD alone. However, current CKD management guidelines (KDIGO, NICE) do not offer specific recommendations for screening or managing cognitive impairment in CKD [44, 45].

Health practitioners should consider screening for cognitive impairment in persons with CKD and aim to delay or manage cognitive impairment by optimizing cardiovascular risk factors, physical activity programmes, cognitive rehabilitation therapies and pharmacological treatment [46]. Furthermore, identifying CKD patients with cognitive impairment may aid prognostication and planning for management of end-stage kidney disease, such as when making decisions on KRT. In a 2019 review of 30 mortality risk calculators in patients starting dialysis, only 7 included cognitive impairment as a variable [47]. Given the results of this review, cognitive impairment should be considered a more significant factor in shared decision-making.

### Suggestions for further research

Further investigation into the association between cognitive impairment and all health outcomes evaluated in this review in persons with non-dialysis CKD is warranted given the current lack of published studies. Future studies should aim to establish causal pathways by controlling for key confounders, particularly cardiovascular risk factors and frailty. Furthermore, studies should analyse whether stratifying participants by age, CKD severity or cognitive impairment severity would affect the risk of poorer health outcomes, thereby identifying the highest risk groups for which interventions could confer the most benefit. More robust cognitive assessment tools should also be utilized where possible, such as the Addenbrooke's Cognitive Examination (ACE-III) which tests a broader range of cognitive domains.

Additionally, more studies should be performed to assess the link between cognitive impairment on self-management ability in CKD and whether this is associated with any change in health outcomes. This will help elucidate if self-management plays a role in mediating the relationship between cognitive impairment and health outcomes.

Finally, this review only included literature on the non-KRT CKD population, thus a systematic review for dialysis and kidney transplant recipients should also be performed.

## CONCLUSION

This review highlights the negative association between cognitive impairment and health outcomes in CKD, including increased all-cause and cardiovascular mortality, greater risk of arrhythmia and cerebrovascular events, lower quality of life, and higher healthcare utilization.

Major limitations include the high heterogeneity of included studies, limited number of studies per outcome and presence of uncontrolled confounding variables, meaning no causal link could be established. Further studies are required to more comprehensively assess all outcomes in this review, especially to explore causal pathways and evaluate the impact of cognitive impairment on self-management ability.

## Supplementary Material

sfaf150_Supplemental_Files

## Data Availability

The data underlying this article are available in the article and in its online Supplementary Material.
