# Supplementary material for: Cognitive impairment and health outcomes in non-dialysis chronic kidney disease: a systematic review and meta-analysis
Source: Clin Kidney J. 2025 May 19;18(6):sfaf150. doi: 10.1093/ckj/sfaf150 (PMC12257935; doi:10.1093/ckj/sfaf150)
Supplement: sfaf150_Supplemental_Files [file sfaf150_supplemental_files.zip › 01 Supplementary Materials_CKJ.docx]

Supplementary Materials

[Supplementary 1 PECO-D framework for the systematic review. 2](#_Toc196231647)

[Supplementary 2 Summary of inclusion and exclusion criteria for the systematic review. 3](#_Toc196231648)

[Supplementary 3 Generic search strategy used. Specific search syntax was altered depending on database used. 4](#_Toc196231649)

[Supplementary 4 Formulas used to calculate pooled hazard ratio and confidence intervals. 5](#_Toc196231650)

[Supplementary 5 R code for meta-analysis of all-cause mortality. 6](#_Toc196231651)

[Supplementary 6 Forest plot showing the results of sensitivity analysis for all-cause mortality, where studies rated as high risk of bias were excluded. 7](#_Toc196231652)

[Supplementary 7 Risk of bias assessment outcomes for all domains in all studies. 8](#_Toc196231653)

[Supplementary 8 List of adjusted variables across all included studies. 9](#_Toc196231654)

Supplementary 1 PECO-D framework for the systematic review.

| **Population** | Patients with chronic kidney disease excluding those in receipt of renal replacement therapy in forms of dialysis or transplantation |
| --- | --- |
| **Exposure** | Diagnosis of dementia or evidence of cognitive impairment on cognitive assessment tools |
| **Control** | Patients without diagnosis of dementia or evidence of cognitive impairment on cognitive assessment tools (although studies without a comparator group may still be considered) |
| **Outcome** | **1**: Self-management (however evaluated) |
|  | **2**: Health outcomes including progression of renal disease (including need for renal replacement therapy, quality of life, hospitalisation, major cardiovascular or cerebrovascular events and mortality |
| **Design** | Cross sectional or cohort studies |

Supplementary 2 Summary of inclusion and exclusion criteria for the systematic review.

|  | INCLUSION | EXCLUSION |
| --- | --- | --- |
| POPULATION | - **Adults (over 18) with chronic kidney disease of any stage** | - **People under 18 years of age.** - **People without CKD** - **People who are on renal replacement therapy (dialysis or transplantation)** |
| EXPOSURE | - **People with a clinical diagnosis of dementia** - **People with scores suggesting dementia or cognitive impairment on cognitive assessment tools or tests for specific cognitive domains** | n/a |
| OUTCOMES | - **Self-management, including but not limited to:**   - Medication or treatment compliance scores   - Patient activation measure (PAM)   - Self-care inventory (SCI)   - Self-management ability scale (SMAS)   - Other patient reported outcome measures (PROM) for ability to self-manage or self-care. - **Health outcomes**   - Renal outcomes, which may include:     - Biochemical measurements of renal function.     - Need for renal replacement therapy.   - Quality of life, with acceptable measures including:     - EQ5D     - Kidney Disease Quality of Life Instrument (KDQOL)     - WHO Quality of Life Questionnaire (WHO-Bref)     - SF-12 or SF-36   - Mortality   - Hospitalisation   - Major cardiovascular events     - Acute myocardial infarction     - Unstable angina     - Heart failure   - Major cerebrovascular events     - Stroke     - Transient ischaemic attack (TIA) | n/a |
| DESIGN | - **Cross-sectional or cohort studies.** | - **Qualitative studies.** - **Case studies and series.** - **Reviews, systematic reviews, or meta-analyses.** - **Conference abstracts prior to 01/01/2020** |

Supplementary 3 Generic search strategy used. Specific search syntax was altered depending on database used.

| Descriptor | Broad Terms |  |
| --- | --- | --- |
| Population  Chronic Kidney Disease | Chronic kidney disease | Chronic kidney disease OR chronic renal disease OR chronic kidney failure OR chronic renal failure OR chronic kidney insufficiency OR chronic renal insufficiency OR chronic kidney impairment OR chronic renal impairment OR chronic kidney dysfunction OR chronic renal dysfunction OR kidney disease OR renal disease OR kidney failure OR renal failure OR kidney insufficiency OR renal insufficiency OR kidney impairment OR renal impairment OR kidney dysfunction OR renal dysfunction OR end-stage renal disease OR end stage renal disease OR end-stage kidney disease OR end stage kidney disease  **MeSH**: Renal Insufficiency, Chronic |
|  | Haemodialysis | "hemodialysis" OR "HD" OR "renal dialysis" OR "dialysis" OR "renal replacement therapy" OR "artificial kidney" OR "renal dialysis therapy" OR "extracorporeal dialysis" OR “haemodialysis” or “haemodiafiltration” OR “hemodiafiltration” |
|  | Peritoneal dialysis | "peritoneal dialysis" OR "PD" OR "abdominal dialysis" OR "home dialysis" OR "CAPD" OR "APD" OR "intraperitoneal dialysis" OR "continuous peritoneal dialysis" |
|  | Renal transplant | "renal transplant" OR "kidney transplant" OR "renal transplantation" OR "kidney transplantation" OR "renal graft" OR "kidney graft" OR "renal organ transplant" OR "kidney organ transplant" |
| Exposure  Cognitive Impairment | Dementia | Dementia OR Alzheimer* OR Senile OR Senile dementia  **MeSH**: Alzheimer Disease; Dementia; Dementia, Vascular |
|  | Cognitive impairment | Cognitive impairment OR cognitive dysfunction OR cognitive deficit OR cognitive deficiency OR cognitive disorder OR cognitive disturbance  **MeSH**: Cognitive Dysfunction |
| Design | Cross-sectional studies | “Cross section*” OR “cross-section*” |
|  | Cohort studies | “cohort” OR “Prospective” OR “longitudinal” |
| Exclusions | Paediatric/ Child/ <18 years |  |
|  | Editorial/ Case Study/ Case Report/ Abstract report |  |
|  | Non-human |  |

Supplementary 4 Formulas used to calculate pooled hazard ratio and confidence intervals.

To calculate the pooled hazard ratio:

The natural logarithm (ln) of each hazard ratio ($ln\left( HR_{i} \right)$) to be combined is calculated.
The standard error for each study’s hazard ratio is calculated ${SE}_{i}=\frac{\ln\left( {CI}_{Upper} \right) - ln({CI}_{Lower})}{2 \cdot1.96}$.
The weight for each study is calculated $w_{i}= \frac{1}{SE_{i}^{2}}$.
The weighted mean of is calculated using $\frac{\sum_{i=1}^{k} w_{i}\cdot ln\left( HR_{i} \right)}{\sum_{i=1}^{k} w_{i}}$.

This is then converted back.

To calculate the pooled hazard ratio:

$${HR}_{combined}= exp\left. \left( \frac{\sum_{i=1}^{k} w_{i}\cdot ln\left( HR_{i} \right)}{\sum_{i=1}^{k} w_{i}} \right. \right)$$

- Where $HR_{i}$ is the hazard ratio from study $i$
- $w_{i}= \frac{1}{SE_{i}^{2}}$ is the weight for each study and ${SE}_{i}=\frac{\ln\left( {CI}_{Upper} \right) - ln({CI}_{Lower})}{2 \cdot1.96}$
- $k$ is the number of studies

To calculate the pooled confidence interval:

$${CI}_{lower}= exp\left. \left( \hat{\mu}- 1.96 \cdot{SE}_{\hat{\mu}} \right. \right)$$

$${CI}_{upper}= exp\left. \left( \hat{\mu}+ 1.96 \cdot{SE}_{\hat{\mu}} \right. \right)$$

- $\hat{\mu}= \frac{\sum_{i=1}^{k} w_{i}\cdot ln\left( HR_{i} \right)}{\sum_{i=1}^{k} w_{i}}$ is the pooled log hazard ratio
- ${SE}_{\hat{\mu}}= \sqrt{\frac{1}{\sum_{i=1}^{k} w_{i}}}$ is the standard error of the pooled log hazard ratio

Supplementary 5 R code for meta-analysis of all-cause mortality.

**library(meta)**

**# Manually input the data for the studies**

**studies <- data.frame(**

**Study = c("Bai 2018", "Burrows 2022", "Chiu 2022", "Corsonello 20224", "Di Rosa 2020", "Faruque 2013", "Merlino 2024", "Raphael 2012"),**

**HR = c(5.32, 1.7, 0.72, 1.36, 1.64, 2.18, 1.63, 1.42), # Hazard Ratios**

**CI_lower = c(1.95, 1.68, 0.32, 0.76, 0.97, 2.01, 1.5, 1.03), # Lower bound of the 95% CI**

**CI_upper = c(14.49, 1.72, 1.64, 2.44, 2.78, 2.36, 1.76, 1.96) # Upper bound of the 95% CI**

**)**

**# Conduct a meta-analysis using the metagen function**

**meta_analysis <- metagen(**

**TE = log(studies$HR), # Log of Hazard Ratios**

**seTE = (log(studies$CI_upper) - log(studies$CI_lower)) / (2 * 1.96), # Standard Error calculation**

**studlab = studies$Study, # Study labels**

**sm = "HR",**

**common = TRUE, # Combine using fixed effect model**

**random = TRUE, # Combine using random effects model**

method.random.ci = TRUE, # Use Hedges' method for small sample correction

**method.tau = "DL" # DerSimonian-Laird method for tau**

**)**

**# Modify the title for the common (fixed) effects model**

**meta_analysis$text.common <- "Fixed effects model"**

**# Print the summary of the meta-analysis**

**print(meta_analysis)**

**# Create a forest plot with the updated label**

**forest(meta_analysis,**

**backtransf = TRUE,**

**sortvar = studies$HR**

**)**

Supplementary 6 Forest plot showing the results of sensitivity analysis for all-cause mortality, where studies rated as high risk of bias were excluded.

**
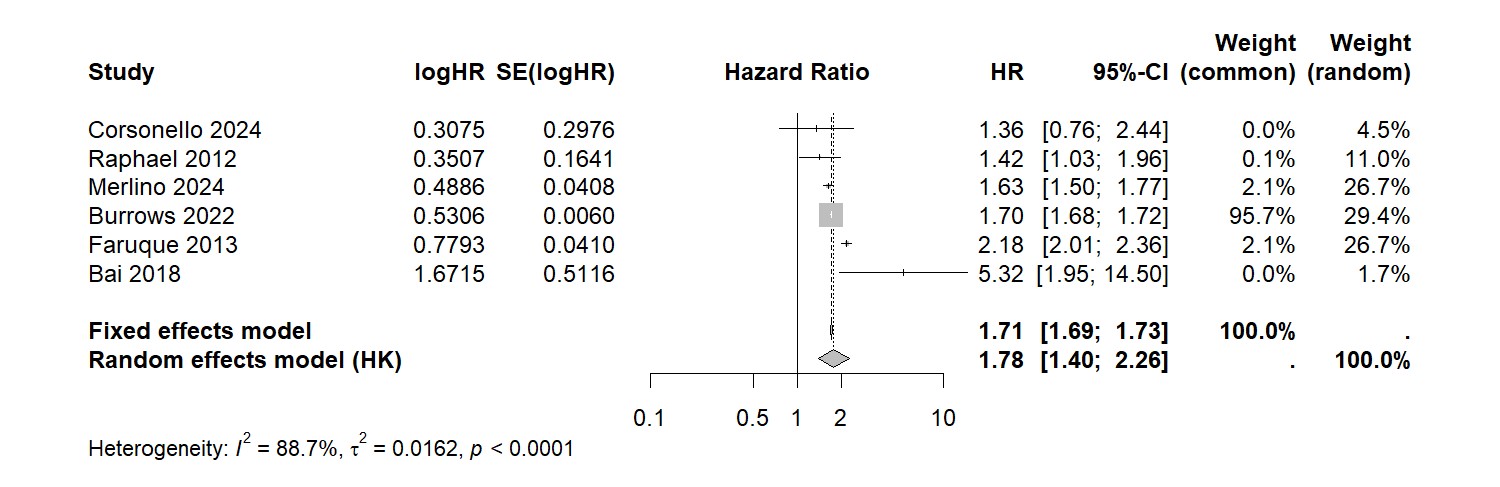
**

Supplementary 7 Risk of bias assessment outcomes for all domains in all studies.

|  | **D1** | **D2** | **D3** | **D4** | **D5** | **D6** | **D7** | **SUM** |
| --- | --- | --- | --- | --- | --- | --- | --- | --- |
| **BAI K** | **Some** | **Low** | **Some** | **Low** | **Low** | **Low** | **Low** | **Some** |
| **BURROWS NR** | **Some** | **Some** | **Some** | **Low** | **Low** | **Low** | **Low** | **Some** |
| **CHIU V** | **Some** | **Low** | **Some** | **Low** | **Some** | **Low** | **Low** | **High** |
| **CORSONELLO** | **Some** | **Low** | **Some** | **Low** | **Low** | **Low** | **Low** | **Some** |
| **DI ROSA M** | **Some** | **Low** | **Some** | **Low** | **Very High** | **Low** | **Low** | **Very High** |
| **FARUQUE L** | **Low** | **Some** | **Some** | **Low** | **Low** | **Low** | **Low** | **Some** |
| **GREINERT R** | **High** | **Low** | **Low** | **Low** | **Low** | **Some** | **Low** | **High** |
| **JAYANTI A** | **Some** | **Low** | **Low** | **Low** | **Low** | **Some** | **Low** | **Some** |
| **KURELLA** | **Some** | **Low** | **Some** | **Low** | **Low** | **Low** | **Low** | **Some** |
| **LU YC** | **Some** | **Low** | **Some** | **Low** | **Low** | **Low** | **Low** | **Some** |
| **MERLINO L** | **Some** | **Some** | **Some** | **Low** | **Low** | **Low** | **Low** | **Some** |
| **RAPHAEL KL** | **Some** | **Some** | **Some** | **Low** | **Low** | **Low** | **Low** | **Some** |
| **SHEETS KM** | **Some** | **Some** | **Some** | **Low** | **Low** | **Low** | **Low** | **Some** |
| **THANCHAROEN** | **Some** | **Low** | **Some** | **Low** | **Low** | **Low** | **Low** | **Some** |

Supplementary 8 List of adjusted variables across all included studies.
