# Supplementary figures and images for: Cognitive impairment and health outcomes in non-dialysis chronic kidney disease: a systematic review and meta-analysis
Source: Clin Kidney J. 2025 May 19;18(6):sfaf150. doi: 10.1093/ckj/sfaf150 (PMC12257935; doi:10.1093/ckj/sfaf150)

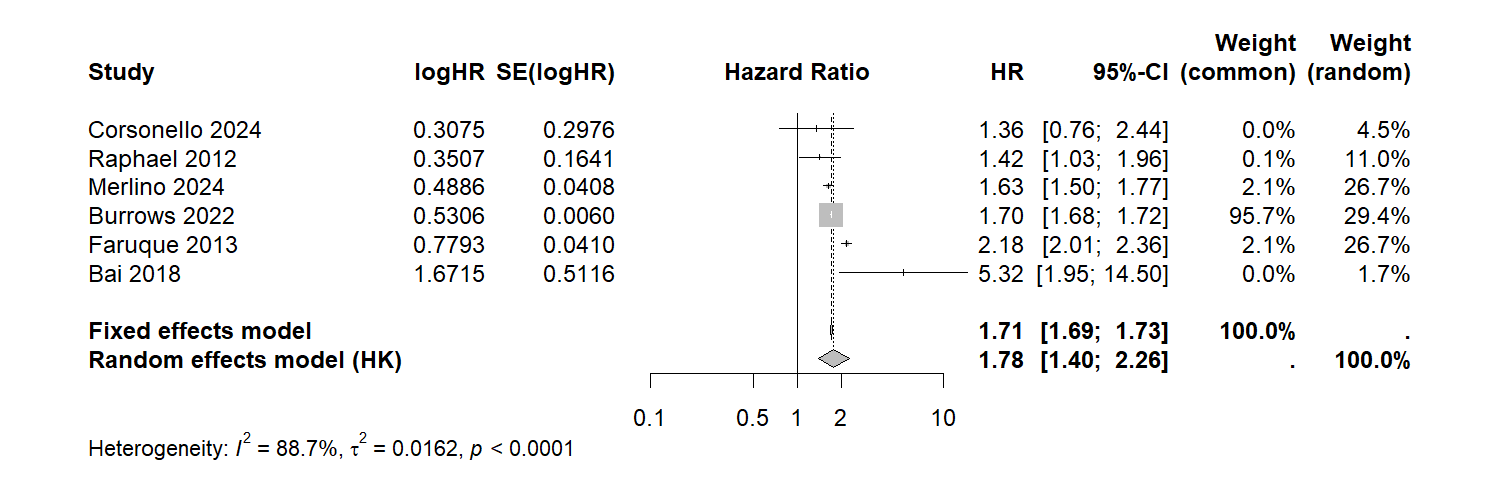

Supplement: sfaf150_Supplemental_Files [file sfaf150_supplemental_files.zip › Supplementary Figure 6 - ACM Sensitivity Analysis.tiff]
